# The Carboxy-Terminal Modulator Protein (CTMP) Regulates Mitochondrial Dynamics

**DOI:** 10.1371/journal.pone.0005471

**Published:** 2009-05-07

**Authors:** Arnaud Parcellier, Lionel A. Tintignac, Elena Zhuravleva, Bettina Dummler, Derek P. Brazil, Debby Hynx, Peter Cron, Susanne Schenk, Vesna Olivieri, Brian A. Hemmings

**Affiliations:** 1 Friedrich Miescher Institute for Biomedical Research, Basel, Switzerland; 2 Interdisciplinary Center of Microscopy, Biozentrum, University of Basel, Basel, Switzerland; Universidade Federal do Rio de Janeiro (UFRJ), Instituto de Biofísica da UFRJ, Brazil

## Abstract

**Background:**

Mitochondria are central to the metabolism of cells and participate in many regulatory and signaling events. They are looked upon as dynamic tubular networks. We showed recently that the Carboxy-Terminal Modulator Protein (CTMP) is a mitochondrial protein that may be released into the cytosol under apoptotic conditions.

**Methodology/Principal Findings:**

Here we report an unexpected function of CTMP in mitochondrial homeostasis. In this study, both full length CTMP, and a CTMP mutant refractory to N-terminal cleavage and leading to an immature protein promote clustering of spherical mitochondria, suggesting a role for CTMP in the fission process. Indeed, cellular depletion of CTMP led to accumulation of swollen and interconnected mitochondria, without affecting the mitochondrial fusion process. Importantly, *in vivo* results support the relevance of these findings, as mitochondria from livers of adult CTMP knockout mice had a similar phenotype to cells depleted of CTMP.

**Conclusions/Significance:**

Together, these results lead us to propose that CTMP has a major function in mitochondrial dynamics and could be involved in the regulation of mitochondrial functions.

## Introduction

Mitochondria are the site of metabolic and survival functions important in organism development, immunity, aging and pathogenesis [1]–[3]. It is becoming clear that these crucial functions within the cell rely on the integrity of the complex double-membrane mitochondria structure that compartmentalizes vastly different enzymatic activities, mainly involved in oxidative phosphorylation [4], the TCA cycle, gluconeogenesis [5], death signal integration [6], [7] and the amplification and transmission of mitochondrial DNA (mtDNA) [8]. Mitochondria within healthy cells are often organized into a dynamic tubular and branched network that undergoes intensive remodeling in response to various stimuli related to cell death [9]–[11] as well as metabolic and developmental processes [12]. The anti-apoptotic Bcl-2 family member Bcl-xL and the antagonist BH3 only proteins Bak/Bax were shown to regulate mitochondrial shape in healthy cells as well as in cells undergoing apoptosis [13], [14]. Thus, the increasing reports of the involvement of signaling proteins in the modulation of mitochondria expose a link between mitochondrial function and dynamics in the regulation of metabolism, cell death, neurotransmission, cell cycle control and development [15].

Studies with yeast led to the identification of the conserved mammalian “mitochondria-shaping” proteins. Profusion proteins, such as the dynamin-related protein mitofusins 1 and 2 (Mfn1 and Mfn2), are integral components of the outer mitochondrial membrane (OMM), essential to mitochondria tethering and fusion [16], [17]. These proteins act together with the optic atrophy protein 1 (OPA1), and an inner mitochondrial membrane (IMM) located dynamin-like GTPase mutated in heritable optical atrophy [18]. Conversely, the dynamin-related protein 1 (Drp1/DNM1) is a cytosolic protein, recruitment of which to the OMM by the anchored fission 1 protein (Fis1p/FIS1) adaptor initiates and controls the fission and distribution of mitochondria in cells [19].

Previously, we identified the Carboxy-Terminal Modulator Protein (CTMP) in a two-hybrid search for PKB/Akt binding partners [20]. CTMP has been shown to inhibit PKB/Akt activation at the plasma membrane in response to various stimuli and also to have tumor suppressor-like functions. This notion was strengthened by the observation that primary glioblastomas exhibit downregulation of CTMP mRNA levels due to promoter hypermethylation [21]. We recently reported the mitochondrial localization of endogenous and exogenous CTMP [22]. CTMP exhibits a dual sub-mitochondrial localization as a membrane-bound pool and a free pool of mature CTMP in the inter-membrane space; it was released from the mitochondria into the cytosol early during apoptosis. CTMP overexpression was associated with an increase in mitochondrial membrane depolarization, caspase-3 and polyADP-ribose polymerase (PARP) cleavage. In contrast, CTMP knockdown resulted in a marked reduction in the loss of mitochondrial membrane potential as well as a decrease in caspase-3 and PARP activation. Mutant CTMP retained in the mitochondria lost its capacity to sensitize cells to apoptosis. Thus, proper maturation of CTMP appears essential for its pro-apoptotic function. Finally, we demonstrated that CTMP delayed PKB/Akt phosphorylation following cell death induction, suggesting that CTMP regulates apoptosis via inhibition of PKB/Akt.

Here we show that compromising Carboxy-Terminal Modulator Protein (CTMP) integrity by preventing its N-terminal cleavage by point mutation or by a knockdown approach affected mitochondrial network organization in cells. CTMP depletion did not affect mitochondria intercomplementation but enhanced the interconnected network, suggesting that CTMP positively influences the mitochondrial fission process, arguing for a potential role of CTMP in regulating mitochondrial functions.

## Results

### A defect in N terminal cleavage of CTMP expression leads to swollen mitochondria

**Figure 1:**
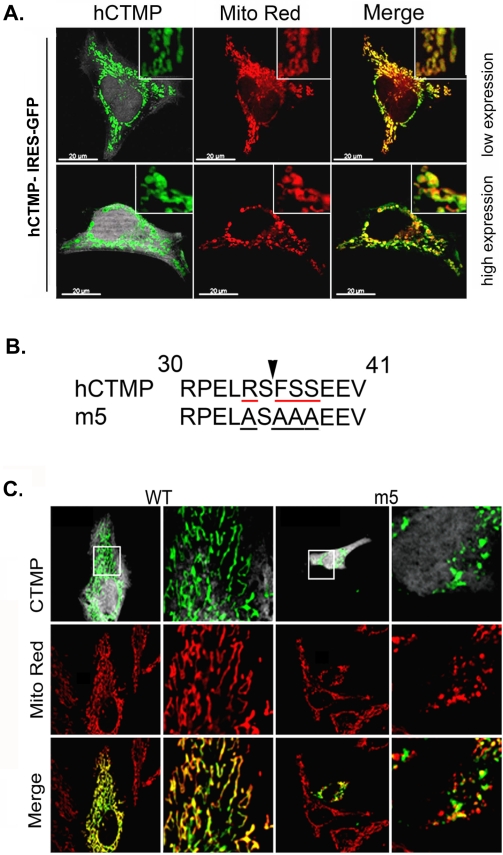
Interfering with CTMP maturation leads to swollen mitochondria. (A) Full length human CTMP tagged with GFP was transfected into HeLa cells at low (upper panels) or high (lower panels) levels of expression. Mitochondria were visualized with Mitotracker Red. Merged fluorescence indicates CTMP-GFP expression in mitochondria. The round appearance of mitochondria is visible in cells with high levels of CTMP expression (lower panels). (B) Amino acid sequence of the R-2 predicted MPP cleavage site (indicated by the arrow) in human CTMP and a CTMP mutant (m5) in which R34, F36 and S37/38 have been mutated to alanine. (C) Twenty-four hours after transfection with CTMP-IRES-GFP or the m5 point mutant, HeLa cells were fixed and stained for CTMP and mitochondria as indicated. A detail of the squared area is shown in the right panel. Representative confocal pictures of three independent experiments are shown.

HeLa cells transfected with full-length CTMP GFP-tagged expressed CTMP in the mitochondria (Fig. 1A). Cells expressing high levels of CTMP induced a change in mitochondrial phenotype in some cells, with more rounded shaped mitochondria evident in these cells (Fig. 1A, lower panels). CTMP contains a conserved N-terminal cleavable mitochondrial localization signal (MLS) and is located almost exclusively in mammalian cell mitochondria. CTMP has been found to be strongly associated with the inner mitochondrial membrane or free in the inter-membrane space [22]. Most MLS are cleaved by a mitochondrial processing peptidase (MPP) that recognizes a special sequence comprising a positive arginine residue at position −2 and/or −10 from the cleavage site [23], [24]. The CTMP sequence displays a highly probable R-2 site at serine 35, surrounded by a hydrophobic residue at +1 and a serine at +2. A CTMP mutant (m5) in which R34, F36 and S37/38 have been mutated to alanine (Fig. 1B) leads to the expression of a non-mature CTMP that still bears the MLS and that cannot be released to the cytosol after apoptosis induction [22]. Similar to full-length CTMP, we observed that over-expression of a CTMP mutant m5 refractory to N-terminal cleavage promoted the formation of rounded, ball-shaped mitochondria, compared with the tubular structures observed in cells transfected with the wild-type protein (Fig. 1C), or untransfected cells (Fig. S1). It should be noted that CTMP subcellular distribution was not affected by its over-expression. These data led us to hypothesize that CTMP may regulate mitochondrial biogenesis.

### Loss of CTMP affects mitochondria morphology

**Figure 2:**
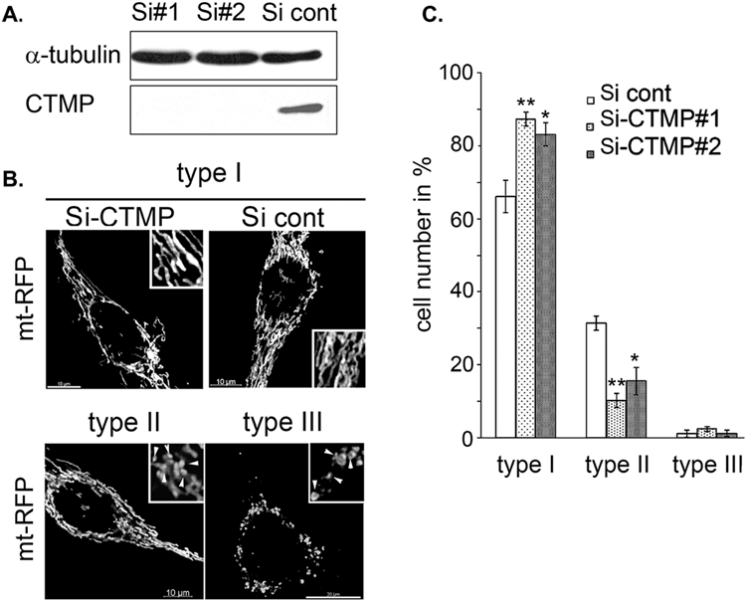
Loss of CTMP affects mitochondria morphology. (A) Immunoblot detection of CTMP using 75 µg of protein lysates extracted from Hela cells 48 h after transfection with control SiRNA (cont) or CTMP SiRNA#1 or #2. (B) Representative confocal picture of mitochondria shape in HeLa cells expressing mt-RFP and treated as in (A). (C) Morphological analysis of mitochondria shape in HeLa cells treated as in (A). For each experiment, at least 200 cells were counted in three distinct fields. Data are means±SEM, *n* = 2. The differences in mean values are statistically significant (Si cont compared to Si-CTMP#1 and Si-CTMP#2) as determined by 1-way ANOVA; * *P*≤0.05, ** *P≤*0.001.

Mitochondrial dynamics is regulated by continuous fusion and fission events. Previous reports indicate that cellular depletion of pro-fission proteins, such as Drp1 or the pro-fusion proteins Mfn-1 and -2, leads to the formation of an interconnected or a fragmented mitochondrial network, respectively [16], [17], [25]. In contrast, overexpression of Mfn-1 or Mfn-2 produces an imbalance in mitochondrial dynamics in a dose-dependent manner and subsequently the perinuclear clustering of the entire mitochondrial network. The putative involvement of CTMP in mitochondrial network rearrangement was further investigated by siRNA-mediated depletion of CTMP in HeLa cells. Efficient and reproducible knockdown of CTMP protein was achieved in cells transfected with two independent CTMP siRNAs (Si#1 and Si#2) compared with the mock siRNA (Si-cont) (Fig. 2A).

**Figure 3:**
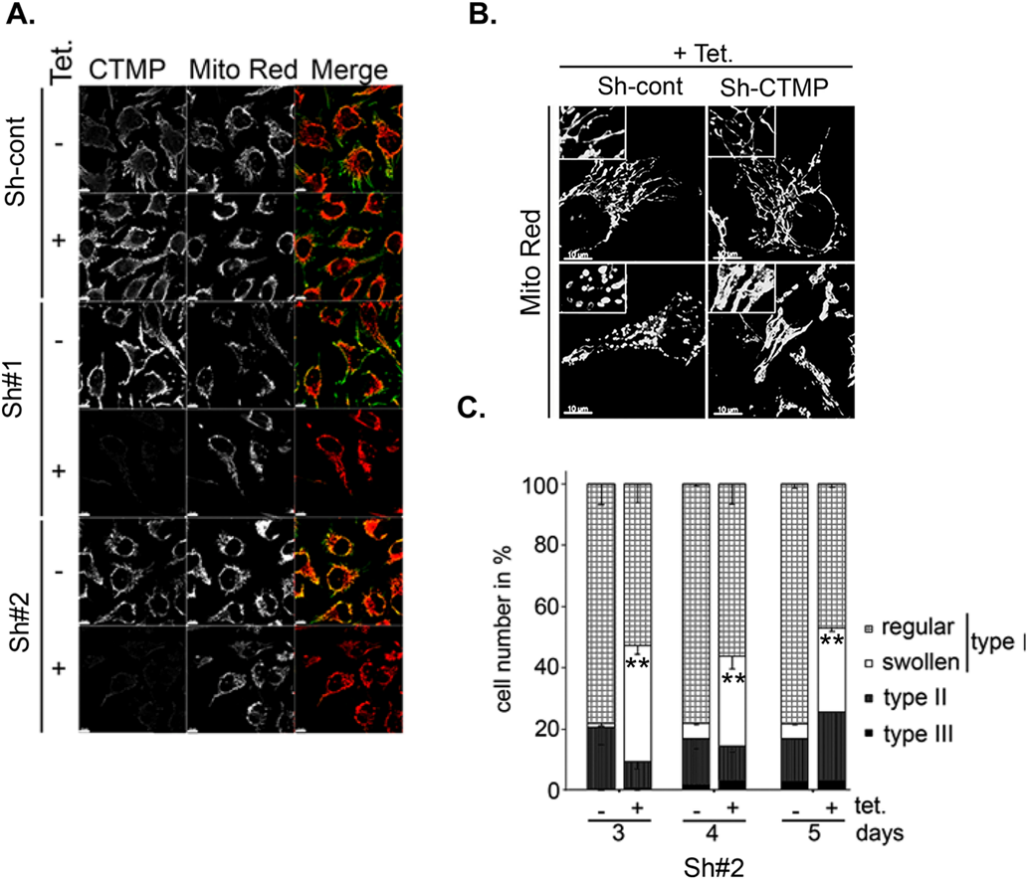
CTMP expression affects mitochondrial network organization. (A) Confocal microscopy of CTMP expression in tetracycline inducible HeLa clones stably expressing pTer plasmid coding for a control short hairpin (Sh-cont) or a short hairpin directed against CTMP (Sh#1 and Sh#2). Cells were visualized by immunofluorescence after 5 days culture in the presence or absence of tetracycline (2 µg/mL), together with the MitoTracker Red. (B) Representative confocal pictures of mitochondrial shape in tetracycline-inducible HeLa clones cultured 5 days in the presence of tetracycline. Mitochondria were visualized with MitoTracker Red. (C) Morphological analysis of mitochondria shape in HeLa Tet-on clones treated as in (A) for 3, 4 or 5 days. For each experiment, at least 200 cells were counted in three distinct fields. Data are means±SEM, *n* = 2. The differences in mean values are statistically significant (Sh#2 minus tetracycline compared to Sh#2 plus tetracycline) as determined by 1-way ANOVA; ** *P≤*0.001.

Cells expressing RFP-labeled mitochondria (mt-RFP) were used to monitor the impact of CTMP depletion on mitochondrial network organization. In these cells, loss of CTMP protein led to a twofold decrease in tubular mitochondrial subpopulation (type II) compared with control cells (Fig. 2B). Although most CTMP-negative cells displayed filamentous mitochondria, detailed confocal examination showed the accumulation of a mixed network of interconnected swollen and thick mitochondria (Fig. 2C; type I Si-CTMP#1 and #2) compared with the Si-cont transfected cells (Fig. 2C; type I Si-cont). To further explore the correlation between CTMP protein depletion and mitochondria remodeling at the single-cell level, tetracycline repressor-expressing HeLa cells were stably transfected with sh-RNA specifically targeting CTMP (CTMP-Sh; targeting a sequence distinct from the previously described Si#1 and Si#2) or control sh-RNA (Fig. 3A). We confirmed that organization of the mitochondria network in CTMP-depleted cells was similar to the previously observed network (Fig. 3B). The population of cells exhibiting a swollen interconnected mitochondrial network was evident 3 days after tetracycline treatment (Fig. 3C). Combined, these results strongly suggest that the modulation of CTMP protein levels and maturation affect mitochondrial shape.

### CTMP depletion does not impair mitochondrial fusion

**Figure 4:**
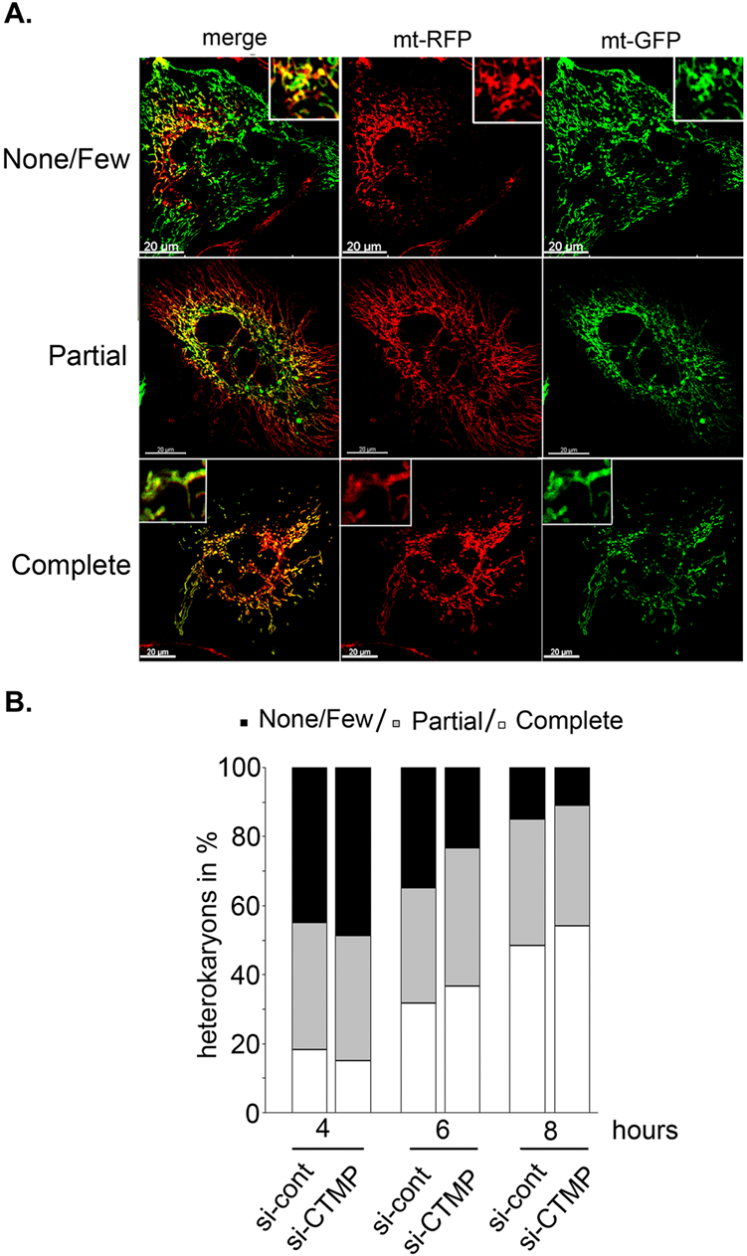
CTMP is not required for mitochondrial fusion. (A) Hela cells expressing mt-RFP and mt-GFP transfected with either siRNA#1 and #2 against CTMP or the siRNA control were fused in 50% PEG1500 for 60 s, washed and fixed at different times (4, 6 and 8 h). (B) Time-course of mitochondrial fusion in HeLa cells treated as in (A). Mitochondrial fusion was measured from 30 randomly selected polykaryons and classified as described in (A), *n* = 2.

To determine whether loss of CTMP function affected mitochondrial fusion or fission, an intermitochondrial complementation assay was carried out using CTMP-depleted cells [26], [27]. Forty-eight hours after transfection with CTMP siRNA (Si#1, Si#2) or the siRNA control (Si-cont), HeLa cells carrying labeled mitochondria (mt-GFP and mt-RFP) were mixed in equal proportions and fused by addition of PEG 1500. Heterokaryons were fixed at the indicated times and mitochondrial fusion kinetics assessed by examination using confocal microscopy of the yellow fluorescence resulting from the mixing of matrix-targeted GFP and RFP mitochondria (Fig. 4A). CTMP-depleted cells completed mitochondria fusion with kinetics comparable to those of control cells (Fig. 4B). These data suggested that CTMP is not critical for the mitochondrial fusion process and further supports the conclusion that the effects of CTMP depletion result from an altered fission process.

### CTMP deletion reveals an extensively interconnected mitochondrial network in mouse liver

**Figure 5:**
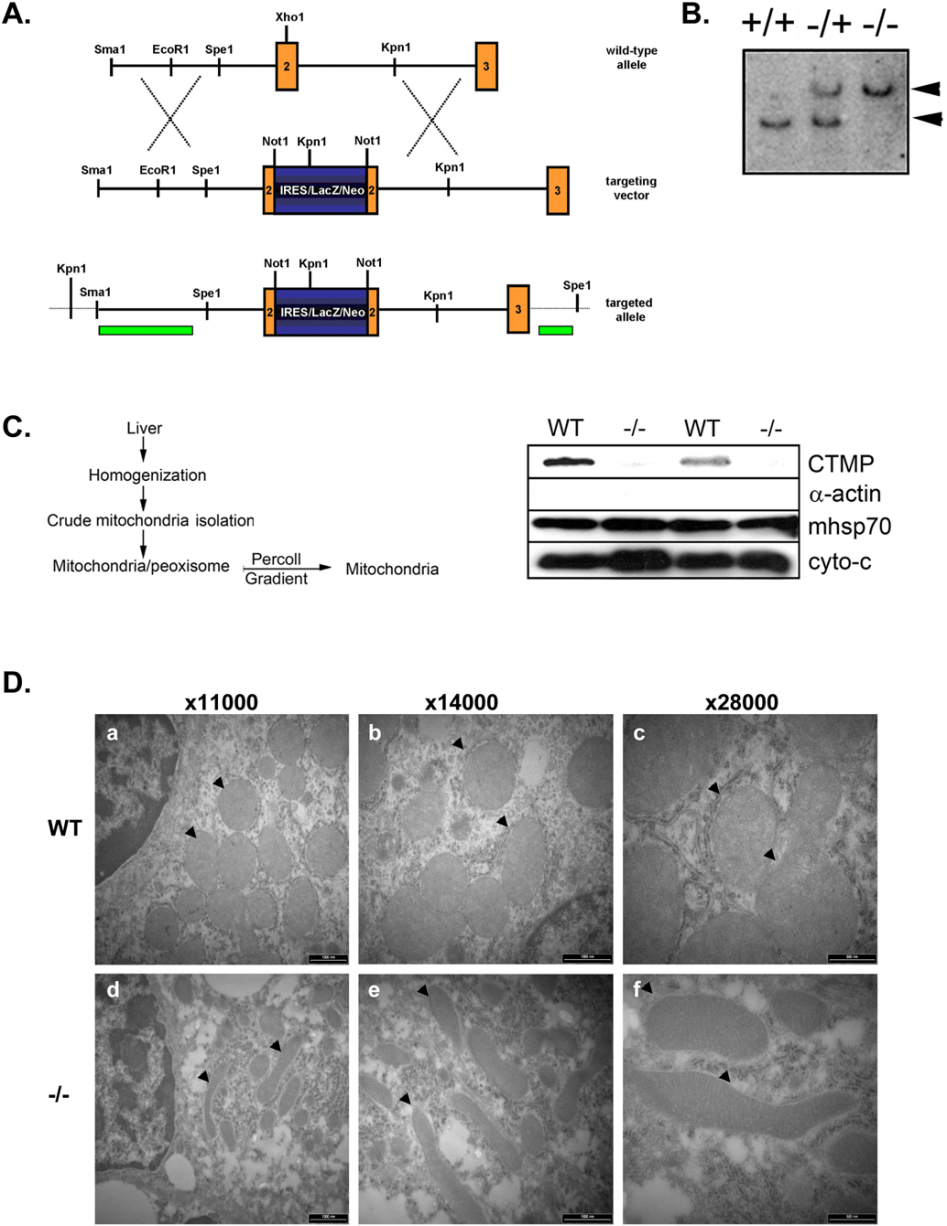
The mitochondrial network is extensively interconnected in CTMP knockout mice liver. (A) Summary of the knockout strategy used to generate CTMP knockout mice. (B) Genotyping of CTMP +/+ (wild-type), +/− (heterozygous) and −/− (knockout) mice. Genomic DNA was digested using Spe1 and probed using a CTMP cDNA fragment. A wild-type band (9 kb, lower band) and a CTMP knockout band (14 kb, upper band) are indicated. (C) Summary of the mitochondria purification strategy outlined in Materials and Methods (left panel). Percoll gradient-isolated liver mitochondria from wild-type (WT) or CTMP knockout (−/−) mice were separated by SDS-PAGE and immunoblotted for α-actin, mHsp70, cytochrome c and CTMP (right panel). (D) Representative electron micrographs of liver mitochondria ultrastructure in (top) wild-type and (bottom) CTMP knockout mice. Arrows indicate normal liver mitochondria (top) and elongated mitochondria (bottom). Representative images of mitochondria shape from different experiments (blind) are shown at different magnifications (×11,000, ×14,000, ×28,000).

We next examined the effect of CTMP deletion on mitochondrial shape at the whole organism level. CTMP knockout mice generated in our lab were viable and fertile and showed no obvious phenotype. A summary of the knockout strategy is given in Fig. 5A, B. We further validated the mitochondrial localization of CTMP in mouse tissue. Immunodetection of CTMP protein in wild-type (WT), heterozygous and homozygous knockout mice showed a correlation between loss of CTMP protein and CTMP allele disruption (data not shown). Mitochondria from WT and CTMP knockout (−/−) mouse livers were purified by differential and density gradient centrifugation (Fig. 5C, left panel). Immunoblot analysis of the collected fractions demonstrated the purity of the mitochondria (cytochrome c and mHsp70) and the absence of cytosolic contaminants (actin). As expected, CTMP immunodetection showed that mouse CTMP protein co-purified with the mitochondrial fraction, as confirmed by the loss of a signal in samples from CTMP knockout mice (Fig. 5C). Electron microscopy of thin liver sections from WT and CTMP knockout mice revealed a correlation between the ablation of CTMP and the appearance of elongated mitochondria (Fig. 5D lower panels), compared with the round and compact mitochondria in the liver of wild-type animals (Fig. 5D upper panels). Accordingly, mitochondria were found to be elongated in hepatocytes isolated from CTMP knockout mice (−/−), compared with round and compact mitochondria observed in hepatocytes of wild-type animals (Fig. S2). Interestingly, loss of CTMP did not interfere with mitochondria biogenesis, since the number of mitochondrial DNA copies measured by real time PCR was the same in brown adipose tissue (Fig. S3A) and in hepatocytes (Fig. S3B) from both WT and knockout animals (−/−). Taken together, these results demonstrate that depletion of CTMP protein impairs mitochondria shape and structure both *in vitro* and *in vivo*.

## Discussion

We provide here the first evidence that CTMP, previously reported to be an inhibitor of PKB/Akt [20], is involved in the modulation of mitochondria homeostasis. We have shown that interference with CTMP expression and/or protein maturation critically affected mitochondria morphology. Loss of cellular CTMP expression led to the establishment of an interconnected mitochondrial network in 40% of cells without affecting cell viability (data not shown). Furthermore, CTMP knockdown appeared to have a second long-term effect leading to the accumulation of swollen mitochondria, either interconnected or tubular. Importantly, such a phenotype has been reported previously in cells depleted for proteins involved in the regulation of the mitochondrial fission process, such as Drp1 or the recently identified Drp1-binding protein MARCH-5 [14], [28]. Mitochondria from CTMP knockdown cells efficiently fuse *in vitro*, suggesting that the observed phenotype, presumably caused by imbalanced fusion/fission, does not result from a dysfunction in mitochondrial fusion. Indeed, we provide evidence that CTMP function in mitochondria is tightly linked to its submitochondrial distribution, where it is found in both soluble and membrane-bound mitochondrial fractions [22]. Expression of a non-cleavable mutant of CTMP (refractory to mitochondrial membrane peptidase cleavage) in HeLa cells promoted dissociation of the mitochondrial network into individual round-shaped, dilated mitochondria.

We recently reported that CTMP is released early from mitochondria into the cytosol upon apoptosis and we demonstrated that CTMP overexpression is associated with an increase in mitochondrial membrane depolarization and the activation of apoptotic markers such as caspase-3 and PARP. Furthermore, we observed that CTMP depletion or a defect in CTMP maturation leads to inhibition of apoptosis. We suggested that CTMP regulates apoptosis via PKB/Akt inhibition since we detected a delay in PKB/Akt phosphorylation in cells overexpressing CTMP in which apoptosis was induced [22]. Similarly, other recent reports suggest that the regulation of OPA1 relies on the activity of a presenilin-associated rhomboid-like protein (PARL), a protease that regulates OPA1 release into the inner mitochondrial space [29]. Furthermore, Cipolat et al. [30] suggested that the loss of this soluble OPA1 species in PARL−/− cells is responsible for their extreme sensitivity to apoptotic stimuli. They further proposed a complex mechanism by which both soluble and inner mitochondrial membrane-anchored OPA1 regulates the tight closure of the mitochondria cristae, preventing massive release of cytochrome c into the inter-mitochondrial space.

Taken together, these observations lead us to hypothesize that accumulation of a premature form of CTMP in the inner mitochondrial membrane (due to inhibition of mitochondrial membrane peptidase cleavage) may be responsible for the observed mitochondrial phenotype. Moreover, *in vitro* characterization of the molecular mechanisms regulating inner mitochondrial membrane dynamics are as yet poorly understood. Surprisingly, a subtle abnormal mitochondrial phenotype in the brain tissue of PKB/Akt knockout mice has been reported recently [31]. These animals display fewer and larger mitochondrial structures, and the authors suggest that PKB/Akt plays a significant role in mitochondrial biogenesis. In addition, PKB/Akt has been shown to translocate to the outer mitochondrial membrane, following plasma membrane activation [32]–[34]. Although the biological significance of the translocation of active PKB/Akt to the mitochondria is not yet clear, it has been reported to be cell-type and stimulus-specific [35], [36]. Thus, CTMP may modulate PKB/Akt activity in a specific subcellular compartment, e.g. the mitochondria or the cytosol, depending on the nature of the stimulus (survival or apoptosis). Further investigation is needed to integrate the direct and/or indirect modulation of PKB/Akt activity in this model and more experiments will be required to address the biological activity of CTMP.

Striking similarities exist between the mitochondria network rearrangement observed in CTMP knockdown cells and those already reported in cells knocked down for the Drp1 fission protein or cells missing a functional OPA1 protein [14], [29]. Nonetheless, the loss of electron absorbance observed in CTMP knockout mice liver mitochondria further supports the involvement of CTMP in the maintenance of inner mitochondrial membrane integrity. Thus, the phenotype observed following CTMP depletion with respect to mitochondrial network rearrangements is less penetrant than those already reported for the key mitochondria-shaping proteins. Moreover, CTMP protein depletion did not affect HeLa cell growth or mitochondrial transmembrane potential measured *in vitro* (data not shown). Most interestingly, it has been shown that CTMP interacts with LETM1, another mitochondrial protein involved in mitochondrial morphology [37]. LETM1 is located in the inner membrane of mitochondria and oligomerized in higher molecular weight complexes [38]. LETM1 has been found to be deleted in Wolf-Hirschorn syndrome (WHS), a complex congenital syndrome characterized by microcephaly, growth and mental retardation, seizures, epilepsy and other associated symptoms [39], [40]. LETM1 is considered as playing a major role in the pathogenesis of seizures. The function of LETM1 in apoptosis, mitochondrial homeostasis and mitochondrial dynamics is well documented, although the different reports drew different conclusions [37], [38], [41]. Thus, it will be very exciting to further investigate the interplay between CTMP and LETM1 in regulating mitochondrial dynamics and functions in future studies. In particular, it would be of interest to explore the phenotype of CTMP knockout mice in the context of Wolf-Hirschorn syndrome.

It is plausible that CTMP mediates its effect by modulating the activity of the key regulators of mitochondrial dynamics and further experiments should address the biological mechanism by which CTMP regulates mitochondrial functions. We have demonstrated already that CTMP exhibits a dual submitochondrial localization. Therefore, the tight association between CTMP protein integrity and maintenance of mitochondria shape observed in this study provides a novel opportunity to investigate the mitochondrial function of CTMP in metabolic regulation.

## Materials and Methods

### Cloning and plasmids construction

All CTMP untagged plasmids used in this study were constructed following PCR amplification of hCTMP cDNA [20] and inserted into the *Bam*HI and *Eco*RI sites of the pcDNA4-IRES-GFP plasmid [42]. CTMP point mutant (m5) was generated by site-directed mutagenesis. To C-terminally tag the CTMP protein, the pcDNA3.1-Myc-RFP plasmid was constructed by subcloning the mRFP1 (monomeric Red Fluorescent Protein 1) cDNA [43] into the *Kpn*I and *Ecor*V sites of the hygromycin resistant vector pcDNA3.1-Myc. The following sequences encoded the CTMP-SiRNA#1 5′-UCGUCAUGACUGCCAAUCU-3′ 5′- AGAUUGGCAGUCAUGACGA-3′ and CTMP Si-RNA#2 5′-CCCAUUUUCUUGACCCAAA-3′, 5′- UUUGGGUCAAG AAAAUGGG-3′ used in this study. The control SiRNAs were directed against the fluorescein protein 5′-UUCUCCGAACGUGUCACGU-3′ and 5′-ACGUGACACGU UCGGAGAA-3′ (Quiagen). To stably induce expression of short hairpins in cells, the CTMP-specific tandem sequences 5′-GATCCCAAGACCCTATACTCAGA GGCGTTCAAGAGACGCCTCTGAGTAGGGTCTTTTTGGAAA-3′ and 5′- AGCTTTT CCAAAAAGACCCTACTCAGAGGCGTCTCTTGAACGCCTCTGAGT ATGGG TCGG-3′ were cloned in the *Bgl*II/*Hind*III sites of pTer vector [44]. The pTer control construct (cont-Sh) used was directed against luciferase as previously described [45] or a scramble sequence 5′GATCCCA GAGACAGCTACCAAGGACTTCAAGAGAGTCCTTGGTAGCTGTCTCTTTTTTGGAAA 3′and 5′-AGCTTTTCCAAAAAAGAGACAGCTACCAAGGACTCTCTTGAAGTCCTTGGTAG CTG TCT CTGG3′. All construct sequences were confirmed using an ABI PRISM 3700 DNA Analyzer (Applied Biosystems).

### Antibodies

CTMP monoclonal antibodies were generated by repeated immunization of BALB/c mice with 50–100 µg of purified full-length His-CTMP protein (produced in *E. coli*), using Stimune (Prionics AG, Schlieren Switzerland) as an adjuvant. Two months after the priming injection, spleenic lymphocytes cells were fused with P3AG8.653 myelanoma cell line (ATCC) and cultured according to standard procedures. After ELISA screening of hybridomas clone supernatants, epitope mapping was carried out for the clone used in this study (52F11) using the GST-CTMP deletion mutant and synthetic polypeptides. The monoclonal anti-CTMP antibody characterized is IgG1. Anti-α-tubulin (YL 1/2) antibody was used as hybridoma supernatants. The commercial mouse anti-mHsp70 (JG1) was from Affinity BioReagents, mouse anti-cytochrome c was from R&D System and rat α-actin was from Santa Cruz Biotechnology.

### Transient and stable transfections

HeLa cells were grown in Dulbecco's Medium (Gibco) supplemented with 10% fetal calf serum. HeLa cell lines stably expressing the tetraycline repressor (HeLa Tet-on) and/or mitochondria-labeled cells (mt-GFP, mt-RFP) were cultured in medium supplemented with 100 ng/mL and 0.4 mg/mL G418 (Sigma), respectively. For transfection, cells were plated in 6-well plates or 10-cm dishes and transfected the following day at 60% confluence using Lipofectamine 2000 following the manufacturer's instruction (Invitrogen). Small inhibitory RNA delivery was achieved with Oligofectamine (Invitrogen). Stable clones expressing CTMP short hairpins or negative controls were selected 48 h after transfection by addition of 0.8 mg/mL Zeocin and positive clones were further maintained in 0.4 mg/mL Zeocin.

### Protein extraction and mitochondria isolation

For whole cell extracts, cells were washed in 1× PBS and resuspended in lysis buffer (50 mM Tris [pH 7.4], 150 mM NaCl, 10% glycerol, 0.5% NP40, 0.5 mM Na-orthovanadate, 50 mM NaF, 80 mM β-glycerophosphate, 10 mM Na-pyrophosphate, 1 mM dithiothreitol, 1 mM EGTA, 10 µg leupeptin/ml and 10 µg aprotinin/ml). Mitochondria isolation was carried out as previously described [41]. Briefly, cells were washed twice in 100 mM sucrose, 1 mM ethylene glycol-bis(β-aminoethyl ether)-tetraacetic acid (EGTA), 20 mM 3-(N-morpholino) propanesulfonic acid (MOPS), pH 7.4 and 1 mg/mL BSA. The pellet was resuspended in the same buffer solution supplemented with 10 mM triethanolamine, 5% (v/v) Percoll, 0.1 mg/mL digitonin for 3 min at 4°C and homogenized with a Potter homogenizer (10 strokes, 1'000 rpm) before being diluted 1/5 in 300 mM sucrose, 1 mM EGTA, 20 mM MOPS, pH 7.4 and 1 mg/mL BSA, and centrifuged at 2'500 *g* for 5 min at 4°C. The supernatant containing mitochondria was collected and centrifuged at 10'000 *g* for 10 min at 4°C to collect mitochondria as a pellet. Isolated mitochondria were washed twice in the same conditions before being resuspended and further processed.

### Western-blotting

For Western blot analysis, protein lysates were prepared by homogenization of various organs in lysis buffer (50 mM Tris-HCl, pH 8.0, 120 mM NaCl, 1% NP-40, 40 mM β-glycerophosphate, 10% glycerol, 4 µM leupeptin, 0.05 mM phenylmethylsulfonyl fluoride, 1 mM benzamidine, 50 mM NaF, 1 mM Na3VO4, 5 mM EDTA, 1 µM Microcystin LR). Homogenates were centrifuged twice (13'000 rpm for 10 min at 4°C) to remove cell debris. Protein concentrations were determined using the Bradford assay. Proteins were separated by 12% or 10% sodium dodecyl sulfate-polyacrylamide gel electrophoresis and then transferred to Immobilon-P polyvinylidene difluoride membranes (Millipore).

### Immunostaining

For immunostaining, cells were grown on coverslips for 24 h following transfection with different plasmids or siRNAs. Where mitochondria were visualized by MitoTracker Red, cells were treated with 300 nM MitoTracker Red CMXRos for 15 min before being washed in PBS and fixed in 3% paraformaldehyde/2% sucrose. Cells were further permeabilized using 0.2% Triton ×100 (3 min at room temperature) before being washed in PBS and incubated together with an appropriate dilution of the primary antibody for 1 h at room temperature in 1% BSA/1% goat serum. This was followed by incubation with secondary antibodies at 1∶100 for 45 min at room temperature. After a final washing, coverslips were mounted in Vectashield medium (Vector lab) and visualized on a laser scanning microscope (Olympus FV500). Confocal images were processed using the Imaris program (Bitplane AG, Zürich, Switzerland) and Photoshop 6.0 (Adobe System Inc).

### Mitochondria intercomplementation

HeLa cells carrying different fluorescent mitochondria (mt-RFP or mt-GFP) were mixed 1/1 and plated on coverslips 24 h after transfection. Mt-GFP HeLa cells were pre-treated for 20 h with 1 mM trichostatin to increase GFP expression levels. After washes in FCS-free DMEM, droplets of 50% PEG 1500 were added directly to cells and aspirated after 45–60 s. After several washes, cells were collected and fixed at the indicated times and processed for immunofluorescence. Heterokaryons were visualized by DNA staining of the nucleus (To-Pro-3 iodide) and/or α-tubulin staining.

### Generation of CTMP knockout mice

For the generation of CTMP mutant mice, a mouse genomic DNA fragment containing exons 2 and 3 was cloned into the pBluescript vector and a *Not*1 site was generated in exon 2. A ∼5-kb IRES-lacZ-Neo cassette was inserted into the *Not*I site, which introduced a translational frame shift. The targeting vector was linearized and electroporated into 129/Ola ES cells. ES cell clones were screened by Southern blotting. DNA was digested with *Spe*I and probed with an external probe. An internal probe was then used on *Kpn*I-digested DNA for further characterization of ES cell clones that were positive for homologous recombination. Correctly targeted ES cells were used to generate chimeras. Male chimeras were mated with wild-type C57BL/6 females to obtain CTMP+/− mice, which were intercrossed to produce CTMP homozygous mutants. Progeny were genotyped for the presence of a targeted allele by multiplex PCR.

### Liver mitochondria isolation

All steps were carried out at 4°C. Mice were housed and terminated according to Swiss legislation. Following termination, freshly dissected liver tissues were immersed and extracted in MSH buffer (pH 7,3) (5 mM HEPES, 70 mM sucrose, 210 mM mannitol, supplemented with 1 mM EDTA), before homogenization in a glass homogenizer (at 500 rpm) in MSH Buffer (supplemented with anti-proteases inhibitors) and centrifugation for 10 min at 800 *g*. The fat coat was removed after centrifugation (10,000 *g* for 10 min at 4°C) and the pellet was manually resuspended in 80 ml of mitochondrial isolation buffer (MSH buffer: 36 µl/ml aprotinin, 5 µl/ml PMSF, 1 µl/ml leupeptin). A crude mitochondrial pellet was isolated by differential centrifugation (3,000 *g*, 10,000 *g* and 9,000 *g*) before being layered on top of a 20-mL Percoll solution (39.3 ml of Percoll, 73.5 ml of 10 mM HEPES, and 13.2 ml of 2.5 M sucrose) and centrifuged at 26,000 rpm for 45 min at 4°C. A pure mitochondria layer was collected below the peroxisome layer and washed twice in mitochondrial isolation buffer before being submitted to protein quantification.

### Transmission electron microscopy

Samples were collected from the same regions of liver (left lobe and median lobe neighboring the gallbladder) for both wild-type [2 females (27,5 and 42 weeks old) and 1 male (42 weeks old)] and CTMP knockout mice [2 females (27,5 and 42 weeks old) and 1 male (42 weeks old)] and immediately fixed for 1 h in 3% paraformaldehyde and 0.5% glutaraldehyde in PBS puffer (pH 7.4), washed twice in PBS and post-fixed for 1 h in 1% osmium tetroxide (OSO4). After dehydration with a graded ethanol series (50–100%) and infiltration in 100% acetone, samples were embedded using an Epoxy-Embedding kit (Epon, FLUKA) for 24 h at 60°C. Thin sections (60–70nm) were obtained on Ultracut (Reichert-Jung) and analyzed on a TEM Moragni 268D (Philips) at 80 kV.

## Supporting Information

Figure S1 (2.26 MB TIF)

Figure S2 (3.10 MB TIF)

Figure S3 (1.17 MB TIF)

Supplementary Text S1 (0.03 MB DOC)
