# Supplementary figures and images for: The Carboxy-Terminal Modulator Protein (CTMP) Regulates Mitochondrial Dynamics
Source: PLoS One. 2009 May 7;4(5):e5471. doi: 10.1371/journal.pone.0005471 (PMC2674955; doi:10.1371/journal.pone.0005471)

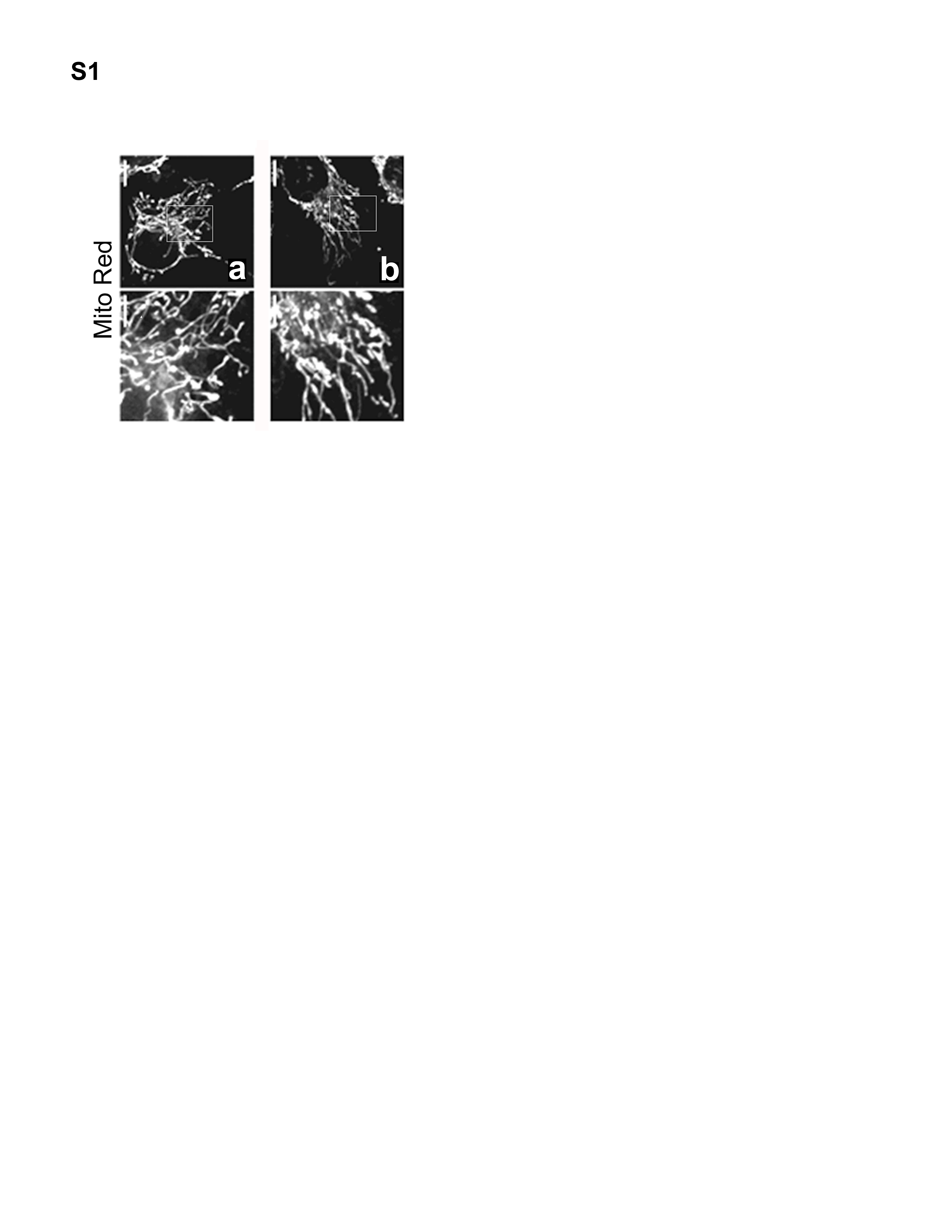

Supplement: Figure S1 — (2.26 MB TIF) [file pone.0005471.s001.tif]

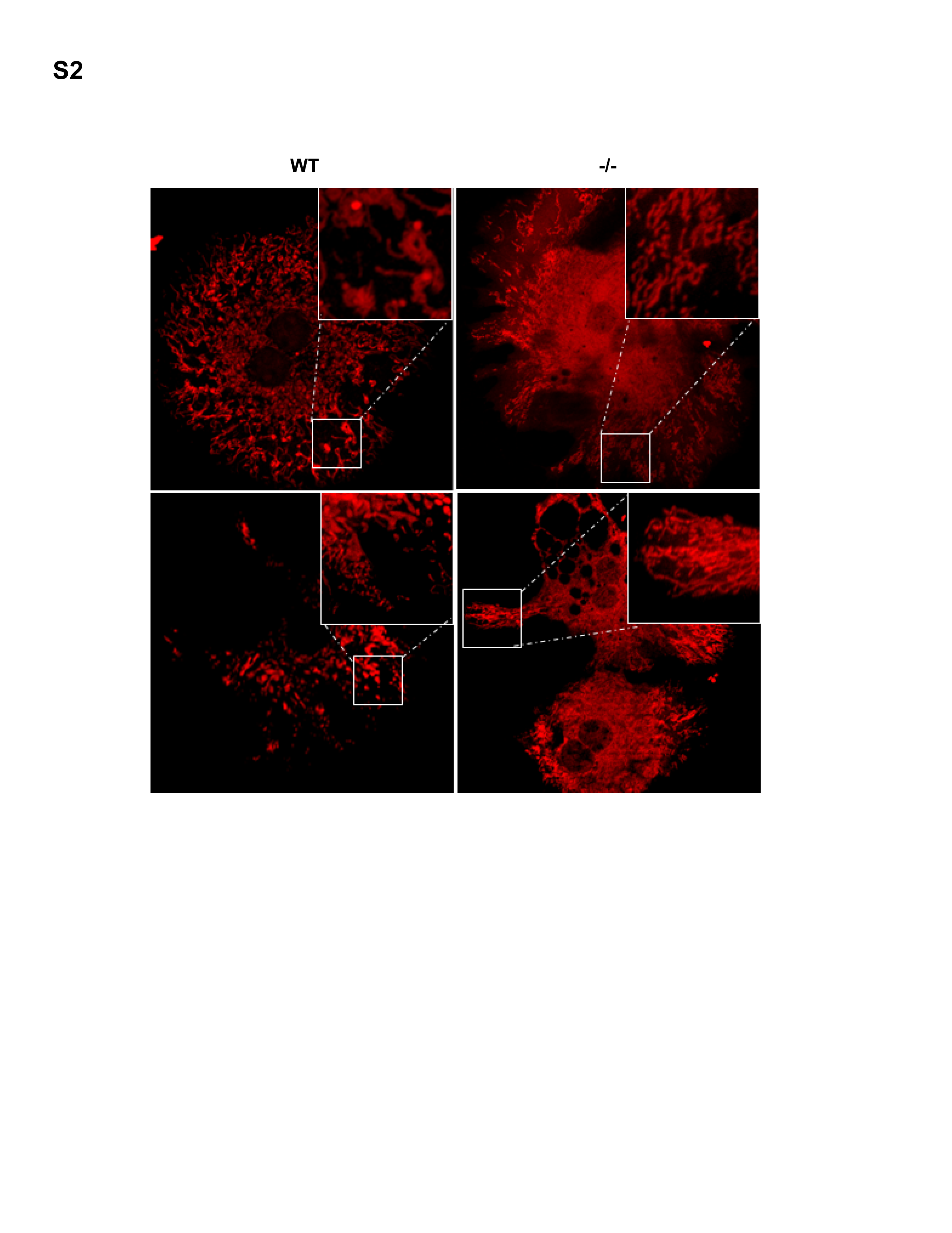

Supplement: Figure S2 — (3.10 MB TIF) [file pone.0005471.s002.tif]

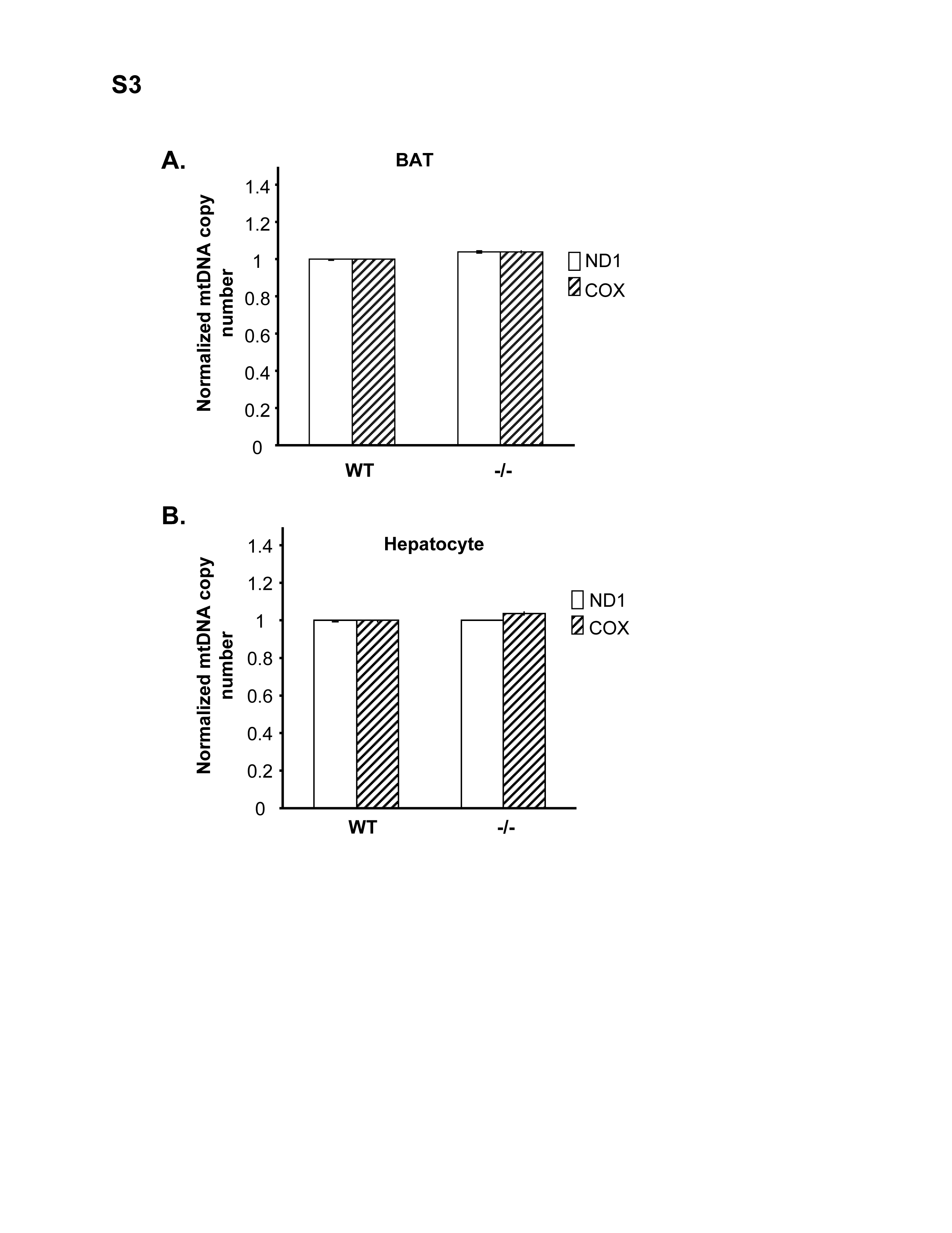

Supplement: Figure S3 — (1.17 MB TIF) [file pone.0005471.s003.tif]
