# Supplementary material for: The Carboxy-Terminal Modulator Protein (CTMP) Regulates Mitochondrial Dynamics
Source: PLoS One. 2009 May 7;4(5):e5471. doi: 10.1371/journal.pone.0005471 (PMC2674955; doi:10.1371/journal.pone.0005471)
Supplement: Supplementary Text S1 — (0.03 MB DOC) [file pone.0005471.s004.doc]

**Supplemental materials and methods**

*Hepatocyte purification*: Mice were perfused via the portal vein and the vena cava was cut to permit outflow of the perfusion solution. The liver was then transferred into a Petri dish containing cold William’s medium E (WME) and the gall bladder was cut. The liver tissue was disintegrated by repeated pipetting and the cell suspension filtered through a 70-mm cell strainer. After washing with ice-cold WME medium, the cells were resuspended in complete WME (supplemented with 100 nm dexamethasone) and plated on collagen-coated coverslips (500 000 cells/well in 12-well plates). After a 4-h incubation at 37^o^C, dead cells were washed away and the viable cells incubated in complete WME medium. The cells were fixed/permeabilized for 15 min in sucrose 2%/PFA 3% and 0.2% Triton X100, respectively, and treated with the mitochondrial red dye MitoTracker.

*RT-PCR Primer sequences (all for mouse proteins) were:* ND1 forward 5’-AGGGTACATA CAACTACGAAAAGGCC, reverse 5’-AGTATTTGGAGTTTGAGGCTCATTC; COX forward 5’-TTGGAGGCTTTGGAAACTGACTTG, reverse 5’-AGTACGGCTGTAATAACTACGGAT CAG. An ABI Prism 7000 detection system (Applied Biosystem) was used in conjunction with the SyBr Green PCR Master Mix (Applied Biosystem) and ABI Prism 7000 SDS Software. Real-time PCR was performed independently at least three times and at least in triplicate. Error bars indicate SD.

**Supplemental figure legends**

***S1:*** Representative confocal images (a, b) of mitochondria shape in healthy HeLa cells. Mitochondria were visualized with Mito Tracker Red.

***S2:*** Representative confocal pictures of mitochondrial shape in hepatocytes from wild-type (WT) or CTMP knockout (-/-) mice. Mitochondria were visualized with MitoTracker Red.

***S3:*** (A) Real-time PCR of total genomic DNA isolated from brown adipose tissue (BAT) and (B) hepatocytes from wild-type (WT) or CTMP knockout (-/-) mice using primers directed against mouse COX and mouse ND1. Mouse GAPDH was used as an internal control. The results are expressed as change in DNA copy number relative to the control. All real-time analyses were performed in triplicate.
